# A preplanning method for stereotactic radiosurgery to improve treatment workflow

**DOI:** 10.1120/jacmp.v17i3.6031

**Published:** 2016-05-08

**Authors:** Kang‐Hyun Ahn, Naim Ozturk, Brett Smith, Konstantin V. Slavin, Matthew Koshy, Bulent Aydogan

**Affiliations:** ^1^ Department of Radiation and Cellular Oncology University of Chicago Chicago IL; ^2^ Department of Radiation Oncology University of Illinois Chicago IL; ^3^ Department of Neurosurgery University of Illinois Chicago IL USA

**Keywords:** SRS, radiosurgery, preplanning, treatment planning, Brainlab

## Abstract

Frame‐based stereotactic radiosurgery (SRS) requires fixation of an invasive head ring to ensure accurate targeting. Minimizing waiting time with a fixed head ring is important for patient comfort and satisfaction. We report a practical preplanning solution for the Brainlab iPlan treatment planning system that reduces waiting time by expediting the planning process on treatment day. A water‐filled anthropomorphic head phantom was used to acquire a surrogate CT image set for preplanning and fused with patient's MRI, which was obtained before the day of treatment. Once an acceptable preplan was obtained, it was saved as a plan template and the phantom image set was removed from the Brainlab database to prevent any confusion and mix‐up of image sets. On the treatment day, the patient's CT and MRI were fused, and the customized beam settings of the preplan template were then applied and optimized. Up to 10‐fold of reduction in treatment plan time was demonstrated by bench testing with multiple planners and a variety of cases. Loading the plan template and fine‐tuning the preconfigured beam settings took only a small fraction of the preplan time to restore the conformity and dose falloff comparable to those of the preplan. For instance, preplan time was 2 hr for a two‐isocenter case, whereas, it took less than 20 min for a less experienced planner to plan it on the day of treatment using the preplan method. The SRS preplanning technique implemented in this study for the Brainlab iPlan treatment planning system offers an opportunity to explore possible beam configurations thoroughly, optimize planning parameters, resolve gantry angle clearance issues, and communicate and address challenges with physicians before the treatment day. Preplanning has been proven to improve plan quality and to improve efficiency in our clinic, especially for multiple‐isocenter and dosimetrically challenging cases.

PACS number(s): 87.53.Ly, 87.55.D‐, 87.55.Gh, 87.55.tm

## I. INTRODUCTION

Intracranial lesions are commonly treated with stereotactic radiosurgery (SRS) where a single fraction of an ablative dose is delivered to the target volume.^1^, ^2^, ^3^ This requires a highly accurate targeting system and traditionally involves an invasive stereotactic head ring to ensure positioning accuracy for the treatment.^4^, ^5^, ^6^ The invasive head ring is fixed prior to the CT simulation and is kept in place until the treatment is delivered. Due to patient discomfort, it is desirable to improve workflow to reduce total procedure time. Linac‐based SRS treatment planning requires a sophisticated arrangement of beams and selection of beam parameters, which is often a challenge even for an experienced planner particularly when demanding dosimetric constraints are in place. This requires substantial time and effort during planning under significant time constraints. The pressure intensifies for newly established SRS programs. Preplanning with available patient images ahead of the head‐ring fixation would greatly improve the workflow on the day of treatment by facilitating the study of best possible treatment plans beforehand.

For most SRS treatments, nonstereotactic patient MRI is acquired a few days before the actual day of treatment, which allows preplanning processes to occur. A few software solutions for preplanning have been developed for Gamma Knife;^7^, ^8^ however, to our knowledge, there is no published preplanning procedure for linac‐based SRS that can work within a commercially available treatment planning system. We report an effective preplanning solution based on an anthropomorphic phantom CT and a patient MRI integrated to work on Brainlab iPlan, a widely used SRS treatment planning system.

## II. MATERIALS AND METHODS

We used Brainlab iPlan RT Image version 4.1 and iPlan RT Dose version 4.5 (Brainlab, Feldkirchen, Germany) installed on a workstation running Microsoft Windows 7 Ultimate. The SRS treatment planning system was configured for our Clinac 2100EX (Varian Medical Systems, Palo Alto, CA) with Millennium 120 multileaf collimator (MLC) and Brainlab conical collimators. Dose distributions were calculated using the pencil beam algorithm, which supports 2.5% accuracy for the simple intracranial geometry.^9^, ^10^, ^11^

**Figure 1:**
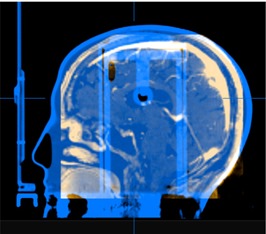
Anthropomorphic head phantom CT (blue) fused with patient MRI (amber) for preplanning.

MRI scans were acquired two to three days before the frame placement. As a surrogate of patient CT, we used a CT image of the RPC anthropomorphic SRS head phantom (Radiological Physics Center, Houston, TX) placed in the Brainlab CT localizer. The phantom is made of a thermoplastic material in the shape of a head of standard dimensions. After importing the phantom CT dataset, localization was performed in the same way as would be done on patient CT. The RPC phantom is filled with water without any heterogeneous tissue modeling. The fusion between the phantom and the patient MRI was manually performed to match the surface coarsely, as displayed in Fig. 1. The coregistered CT‐MRI dataset allowed treatment planning to be performed based on the approved patient‐specific contour sets drawn on the MRI images and examination of the dose distribution calculated on the phantom CT in iPlan RT Dose software.

Brainlab organizes imported DICOM files in a vendor‐specific xBrain format. To avoid potential data corruption in the xBrain database, we devised a file management scheme that maintained only a single instance of a CT scan at any given time, either of the phantom or the patient, within the same patient dataset. Once the contours were entered and approved by an attending physician on the MRI images, the entire xBrain folder of the patient was copied to a separate folder before we proceeded with importing the phantom CT. After an acceptable plan was configured, it was saved as a template that contained all the plan parameters including cone diameters, beam weightings, margins, collimator angles, gantry angles, and table angles. This allowed us to retrieve the plan information even after the preplanned xBrain folder was replaced with the initial dataset that had the patient MRI with approved contours. The template did not include MLC positions, which were handled in the fine‐tuning procedure, as described below.

**Figure 2:**
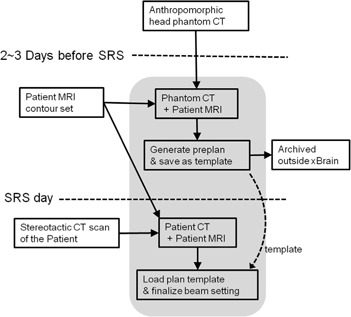
SRS preplanning workflow. Shaded area represents the works performed within the xBrain database.

On the day of SRS treatment, we imported the patient CT into the xBrain dataset free of the phantom image and its usage logs. Planning was then repeated with the patient CT using the beam settings retrieved from the template. The replacement of phantom CT with the patient CT induced a slight change in dose distribution. This required a few minutes of fine‐tuning that involved adjustment of weighting between the beams, beam isocenter position for the cone (usually within±mm), and one or two iterations of MLC leaf position adjustments. The preplanning workflow is summarized in Fig. 2.

Bench testing was performed to measure treatment plan times for nine conformal beam and seven circular arc cases. Preplans were created to achieve the minimum acceptable criteria. Planner 1 and planner 2 created preplans and also performed treatment planning using patient CT images a few days later. Planner 3 and a less‐experienced planner (planner 4) were asked to perform treatment planning using the preplans created by the planners 1 and 2. When calculating and reporting the planning time, we excluded the routine tasks such as importing, localizing, CT registration, and reviewing the contour. Test cases included in this study ranged in planning difficulty from spherical tumors that were away from organs at risk (OAR) to relatively eccentric target volumes in the immediate vicinity of OARs. Institutional Review Board approval was obtained for this study.

## III. RESULTS

**Figure 3:**
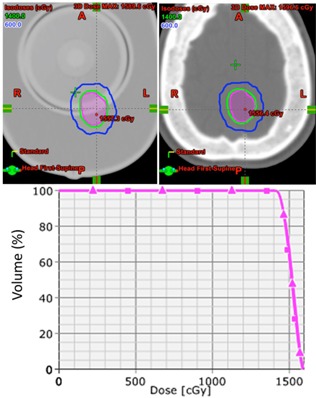
Preplan (top left) generated for a 2 cm target volume (magenta) in 25 min using 10 conformal beams. Treatment plan (top right) generated in less than 5 min using patient CT with the preplan template. DVH (bottom) of the preplan (square) and the template‐generated plan (triangle) demonstrates a good agreement.

Figure 3 compares dose distributions on a single axial slice and the DVH for the target for an occipital meningioma case. A preplan was generated for the phantom CT in 25 min to satisfy <120% homogeneity, 100% coverage, and < 2.0 conformity index (CI, ICRU 62 definition)^(12)^ using 10 conformal beams. Using the preplan, a comparable patient plan was obtained in 5 min. The shape of 14 Gy and 6 Gy axial isodose lines and DVH lines shows that the dose homogeneity, coverage, and conformity achieved in the phantom CT were closely reproduced in the patient CT. The target shapes were originally segmented in the MRI and underwent a slight change by being resampled in each of the CT, as shown in Fig. 3.

**Figure 4:**
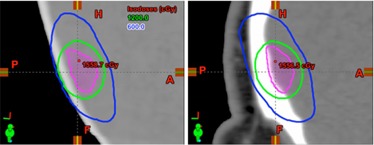
Sagittal isodose lines of the preplan (left) for an elongated target volume (magenta); (right) the corresponding patient plan generated using the preplan. The preplan evaluated and approved by the physician was closely reproduced on the day of treatment using the preplan beam configuration in less than 5 min.

Figure 4 shows sagittal dose distributions of a preplan and a template‐generated plan for an oblong target volume. The preplan was generated in 90 min to satisfy <130% homogeneity, 99.8% coverage, and a CI of 3.1 using four circular arcs. The target volume had an elongated extension (∼1.4 cm) toward the superoposterior direction, and the arcs were arranged to conform the dose distributions to this particular shape. Again, application of the preplanned template on the patient CT achieved the same dosimetric criteria in less than 3 min. Although the outer contour and the bony anatomy were not perfectly modeled by the phantom CT, the overall shape of the isodose lines in the patient CT was similar to that of the preplan.

The reduction of planning time was evaluated with three planners (planner 1, 2, 3) by measuring their preplan and treatment plan times. Table 1 lists results of the bench testing for 11 cases. Planner 1 generated five preplans to be tested by planner 1 and 2. Planner 2 generated six preplans to be tested by planner 2 and 3. The preplan time ranged 20–120 min depending on the number, size, shape, and location of tumors to achieve 100% coverage with <130% maximum dose and CI ranging from 1.5 to 3. Acceptable treatment plans, which satisfied most of the dosimetric parameters achieved by the preplans, were obtained in less than 20 min using the preplan method. For instance, the preplan for patient 3 with four circular arcs took 90 min to optimize for a 0.8 cc PTV for the planner 1 who achieved a 99.8% PTV coverage with the prescription dose, 130% maximum dose, and 3.1 CI. When this case was benchmarked, both planner 1 and 2 generated treatment plans with almost identical preplan parameters in 3 min.

**Table 1:** Preplan and treatment plan results for 10 patients.

*Patient*	*PTV Volume*	*# of Beams*	*Plans* ^a^	*%PTV Coverage*	*%Max Dose*	*Conformity Index*	*Time (min)*
1	1.0 cc	4^b^	PP1	100	130	1.98	20
			TP1/TP2	99.7 / 99.9	130 / 131	1.91 / 2.01	2 / 3
2	3.3 cc	5^b^	PP1	100	130	1.98	20
			TP1/TP2	99.8 / 99.7	130 / 131.3	1.94 / 1.95	2 / 3
3	4.3 cc	10^c^	PP1	100	115	1.5	25
			TP1/TP2	100 / 99.8	116/ 117	1.52 / 1.55	5 / 6
4	3.5 cc	12^c^	PP1	100	114	1.49	30
			TP1/TP2	100 / 100	115 / 116	1.55 / 1.55	5 / 5
5	0.8 cc	4^b^	PP1	99.8	130	3.1	90
			TP1/TP2	99.7 / 99.8	131 / 133	3.0 / 3.1	3 / 3
6	4.4 cc	12^c^	PP2	99.7	126	2.01	41
			TP2/TP3	99.6 / 99.8	124 / 124	1.98 / 2.07	3 / 5
7	1.0 cc	5^b^	PP2	99.8	118	1.54	38
			PP2/TP3	99.6 / 99.9	120 / 119	1.55 / 1.56	3 / 2
8	3.3 cc	11^c^	PP2	99.8	116	1.21	35
			PP2/TP3	99.9 / 99.8	118 / 121	1.22 / 1.24	4 / 3
9	0.75 cc	10^c^	PP2	99.8	116	2.28	44
			PP2/TP3	99.8 / 99.7	117 / 118	2.49 / 2.59	6 / 10
10	0.24 cc	4^b^	PP2	99.6	123	2.29	
			PP2/TP3	99.5 / 99.5	125 / 125	2.22 / 2.26	120
10	6.4 cc	11^c^	PP2	99.8	124	1.51	15 / 20
			PP2/TP3	99.9 / 99.9	127 / 127	1.62 / 1.64	

a PP1: Preplan by planner 1; PP2: Preplan by planner 2; TP1/TP2: Treatment plans by planner 1 and planner 2; TP2/TP3: Treatment plans by planner 2 and planner 3.

b Circular arcs.

c Conformal beams.

Patient 10 with a 0.24 cc tumor treated with cones and a 6.4 cc tumor treated with conformal beams presented a greater challenge. Planner 2 prepared a preplan in 120 min, which satisfied the plan criteria provided by the radiation oncologist. The same planner reproduced corresponding patient‐specific treatment plan with similar dosimetric criteria using preplans in 15 min on the day of treatment. The third planner was able to obtain a treatment plan almost identical to preplan in less than 20 min compare to 120 min of preplan times. The proposed method not only ensured exhaustive search in consultation with the radiation oncologist for the best possible plan, but also improved the workflow and the patient comfort on the day of treatment.

Table 2 displays information of a patient with metastatic brain lesions treated in our clinic. The patient had five lesions, which included the post‐op bed, as well as tumors in the right internal capsule, pons, left cerebellum, and in the left occipital lobe. The preplan procedure took 3 hr for the planner 2, which included the identification of the proper arc angles for each isocenter to avoid overlapping and to minimize dose to critical structures, as well as the cross‐dose contribution. An initial planning consultation, several modifications, and review were done with the attending physician to ensure the plan met his criteria. On the day of treatment, the same planner used the preplan parameters to generate a similar plan for each isocenter successfully with some minor modification in less than 1 hr. A less‐experienced planner, planner 4, was asked to generate a five‐isocenter plan using the preplan done previously. He was able to generate plans that were very similar to the preplan approved by the attending physician earlier in 65 min compared to 3 hr, which was the time spent by an experienced planner during preplanning process.

**Figure 5:**
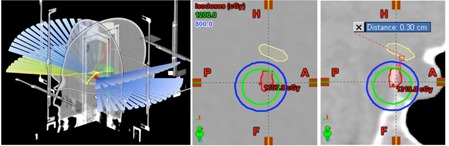
Preplan beam configuration (left) for a target volume (red) 3 mm inferior to the optic chiasm (yellow). Sagittal isodose lines of the preplan (center). The corresponding patient plan (right). The beam isocenter was shifted from the target center to secure >1 mm distance between the 8 Gy isodose line and the optic chiasm.

Figure 5 shows the beam configuration and the dose distribution comparison of the preplan and treatment plan generated using the preplan in a fraction of the preplan time for a meningioma with sellar invasion. The target volume was 3 mm inferior to the optic chiasm, which prioritized protecting this OAR with a maximum dose less than 8 Gy, or 67% of the prescription dose. During preplanning, the beam isocenter was shifted posteriorly and inferiorly from the center of the target volume to ensure more than 1 mm separation between the optic chiasm and the 8 Gy isodose line. The preplan achieved 92% coverage with the CI of 6.0 and <109% homogeneity. When the preplan setting was applied, a treatment plan with 90% coverage, CI of 6.1, and <110% dose inhomogeneity was achieved using the patient CT in a fraction of time spent for the preplanning,

**Table 2:** Preplan and treatment plan results for a patient with five metastatic brain lesions.

*PTV Volume*	*# of Beams*	*Plans* ^a^	*%PTV Coverage*	*%Max Dose*	*Conformity Index*	*Time (min)*
0.26 cc	4^b^	PP2	99.7	110	2.25	
		TP2/TP4	99.5/ 99.4	107 / 110	2.18 / 1.22	
0.49 cc	3^b^	PP2	99.7	114	2.65	
		TP2/TP4	99.9 / 99.8	112 / 115	2.55 / 2.61	
0.64 cc	10^c^	PP2	99.9	117	1.77	180
		TP2/TP4	99.9 / 99.9	115 / 118	1.65 / 1.75	60 / 65
1.0 cc	10^c^	PP2	99.7	118	1.71	
		TP2/TP4	99.6/ 99.5	115 / 117	1.66 / 1.73	
8.41 cc	11^c^	PP2	99.8	119	1.79	
		TP2/TP4	100 / 99.8	117/ 120	1.74 / 1.8	

a PP2: Preplan by planner 2; TP2/TP4: Treatment plan by planner 2 and planner 4.

b Circular arcs.

c Conformal beams.

## IV. DISCUSSION

Optimization of the plan quality and efficiency of SRS process, while minimizing the patient waiting time, with an invasive head ring on in the frame‐based SRS is highly desirable for delivering the best possible plan and for patient satisfaction. SRS preplanning performed prior to the day of treatment may allow the planner to thoroughly address planning challenges specific to each patient without the time constraints experienced on the day of treatment. Furthermore, preplan method allows the planners to familiarize themselves with the case, discuss any challenging issues with the treating physicians, and consult with neurosurgeons few days before treatment. This may be particularly valuable for the new SRS programs and/or inexperienced planners.

Frameless SRS^13^, ^14^ allows ample time for planning, but it imposes different challenges in implementation, and may not be viable or acceptable to every practitioner. Brainlab iPlan, although it is widely used for framed‐based SRS treatment planning,^15^, ^16^, ^17^ does not provide a working solution for preplanning since CT image sets with the frame affixed to the patient are not available until the day of treatment. We developed an efficient, safe, and reliable preplanning procedure for frame‐based SRS within the framework of Brainlab iPlan system.

We used a water‐filled anthropomorphic phantom to provide a CT image for the iPlan system to perform planning with the contour sets defined on the patient MRI. In principle, an anonymous head CT scan of a patient could be used as a phantom to model the internal anatomy and electron density. However, this involves a potential safety issue of mistakenly switching the CT images of phantom with the actual patient because the anonymous images would not easily be distinguished from that of the patient to be treated. The simplified internal anatomy in the anthropomorphic phantom served as a failsafe identifier with negligible dosimetric deviations, as indicated in our results.

One of the potential limitations of the study design may be the possible dosimetric variations and heterogeneity correction associated with the use of anthropomorphic phantom. Notwithstanding the differences between the phantom and patient, our results displayed in 1, 2 and 3, 4, 5 demonstrate the dosimetric differences were negligible. For instance, there were some minor visual variations near the skin in the preplan as shown in Fig. 4. Nevertheless, its dosimetric effect was negligible in the treatment plan as expected due to the simple anatomy of human cranium, which simplifies the problem to mainly having different effective depths of treatment. Modern treatment planning algorithms can easily address these differences by scaling during plan normalization. Further, replicating the preplan with less than 2% difference within a few minutes with the proposed method justifies the use of anthropomorphic phantom.

Our preplanning approach successfully demonstrated dosimetric reproducibility with considerable reduction in treatment plan time. The results of bench testing in Table 1 validate the reproduction of dosimetric criteria with multiple planners and variety of cases. In particular, planner 2 spent substantially longer time than planner 1, and improved the dosimetric parameters to some extent. Spending longer time in preplanning, however, does not always produce proportionately improved treatment planning results. In fact, finding out how far we can push a treatment plan to improve dosimetrically is a grey area and may take an indefinite time and still result in a suboptimal solution. With preplanning, we can work on these open‐ended problems before the placement of the invasive head frame to improve and facilitate patient care. With the patient‐specific template that can readily restore the preplanning dosimetric results on patient CT, the SRS workflow can be greatly improved on the treatment day. Furthermore, preconfigured beam settings obtained with the preplanning method may also provide an opportunity to confirm equipment clearances for particular orientations of the gantry and couch before the treatment day, which would not only improve the workflow but also result in substantial time saving on the day of treatment.

Another compelling reason to practice preplanning is to perform feasibility study for SRS cases with extremely difficult dosimetric constraints. Figure 5 presents a target volume in close proximity of the optic chiasm where its protection may necessitate a significant compromise in either target volume coverage or conformity. To ensure that optic chiasm receives less than 8 Gy dose, target volume was covered with a large conformity index in the preplan, which was reliably reproduced in the actual treatment planning. In this case, the preplan served as a useful feasibility study tool to analyze the trade‐off between the target dosimetry and the OAR protection. It is critical to evaluate the required dosimetric adaptation prior to the treatment day if the SRS approach is indicated to be questionable. With the proposed preplan method, not only will the treatment team have ample time to discuss and look for an optimal plan that minimizes any compromises, but also they may consider aborting the treatment before ring placement if obtaining an acceptable SRS plan does not seem feasible.

The preplanning method described in this study can be further refined by synthesizing a CT from the nonstereotactic patient MRI. Multiple groups have been extensively studied the use of MRI alone as the basis for treatment planning.^18^, ^19^, ^20^ Most of the studies make a substantial effort to extract accurate CT numbers and to address potential geometric distortions. However, the results from our single‐phantom surrogate approach indicate that even a homogeneous synthetic CT with crude distortion correction could provide a clinically working solution for intracranial SRS preplanning where the dosimetric effect of heterogeneity is negligible.

## V. CONCLUSIONS

In the frame‐based SRS, a plan needs to be generated on treatment day while the patient is waiting with an invasive head ring attached, and this could present a challenge in patient care, particularly if the case involves stringent dosimetric constraints. Our preplanning technique effectively reduces planning time considerably and, more importantly, enables us to exhaustively explore acceptable beam configurations for optimizing a plan before the treatment day. This can be particularly valuable for centers that are new to SRS by offering an opportunity for case‐specific training — that is to say, searching thoroughly for optimal planning parameters, communicating challenges with physicians, and addressing gantry angle clearance issues before the treatment day. Further, the advantages of preplanning can be imperative particularly for multiple‐isocenter and dosimetrically challenging cases.

## COPYRIGHT

This work is licensed under a Creative Commons Attribution 4.0 International License.
